# A Self-Healing Adhesive Exploiting Hydrogen-Bonding Interactions and Aromatic Disulfide Units in Energetic Composites

**DOI:** 10.3390/polym18212608

**Published:** 2026-10-22

**Authors:** Huan Zhao, Xiuduo Song, Weilu Yang, Yiwen Hu, Jiangfeng Pei, Bofan Shen, Zhiyu Chen, Jian Kang

**Affiliations:** 1 Xi’an Modern Chemistry Research Institute, Xi’an 710065, China; 2 State Key Laboratory of Advanced Polymer Materials, Polymer Research Institute of Sichuan University, Sichuan University, Chengdu 610065, China

**Keywords:** self-healing adhesive, dynamic disulfide bond, hydrogen bond, energetic composites

## Abstract

Energetic composites with high solid content are prone to microcracking and interfacial debonding, which can compromise mechanical integrity and service reliability. To address these long-standing durability challenges, herein, a self-healing polyurethane with hydrogen bonds and disulfide bonds (PUHD) was designed by integrating hydrogen-bond reorganization with dynamic disulfide-bond exchange. PUHD was synthesized using polytetramethylene ether glycol (PTMEG) as the soft segment and isophorone diisocyanate (IPDI) and 4,4′-dihydroxydiphenyl disulfide (DHDPS) as hard-segment components. FT-IR, Raman, XRD, GPC, and DSC analyses confirmed the successful construction of a predominantly amorphous dual dynamic network with suitable chain mobility and thermal stability for energetic-composite applications. The neat PUHD exhibited a hardness of 0.94 MPa and a storage modulus of 3.15 MPa at 20 °C and 5 Hz; surface cracks disappeared within 24 h at ambient temperature, and the storage-modulus recovery reached approximately 97%. In sample filler-containing composites, increasing solid content enhanced hardness and modulus but restricted polymer-chain mobility, thereby slowing crack repair and reducing healing recovery. In nitrocellulose/nitroglycerin (NC/NG)-containing composites, the visible crack closure could be achieved within 24 h, while maintaining useful mechanical properties after self-healing. Notably, JQ-e showed the highest initial hardness of 2.69 MPa and retained 2.19 MPa after healing. These findings collectively demonstrate that the synergistic dynamic network not only enables efficient room-temperature crack repair but also preserves adequate mechanical reinforcement, thus offering a viable pathway toward more reliable energetic-composite formulations.

## 1. Introduction

Energetic composites are key functional materials for propulsion and defense applications, where high energy output, structural integrity, and service reliability must be simultaneously maintained [1,2]. These materials generally consist of energetic crystals or solid fillers embedded in a polymeric adhesive matrix. The adhesive phase provides particle coating, interfacial bonding, shape retention, and mechanical support, thereby influencing processing behavior, mechanical response, energy release, and environmental durability [3]. In particular, the binder matrix plays a critical role in dissipating stress and preventing catastrophic failure under both quasi-static and dynamic loading conditions. However, modern formulations often require high solid content to improve energy density, which reduces the continuity of the adhesive phase and increases the probability of stress concentration, microcracking, and interfacial debonding during processing, storage, or mechanical loading [4,5]. Such damage can weaken mechanical integrity and may further increase safety risks associated with unstable combustion or accidental ignition [6].

Therefore, endowing the adhesive matrix with intrinsic self-healing capability is a promising route to improving the damage tolerance of energetic composites [7]. Self-healing polyurethanes are particularly attractive because their segmented structures allow mechanical properties, chain mobility, and dynamic interactions to be adjusted through molecular design [8]. Typical reversible motifs include hydrogen bonds, π-π interactions, metal–ligand coordination, and dynamic covalent bonds such as Diels–Alder adducts, imine bonds, borate ester bonds, and disulfide bonds [9]. Among these, hydrogen bonds offer the advantage of rapid reversibility under mild conditions, whereas disulfide bonds provide covalent reconnection that can restore network topology. Nevertheless, a single healing mechanism often cannot simultaneously provide rapid crack repair and sufficient mechanical recovery [10]. Hydrogen bonds can reversibly dissociate and reform under mild conditions, allowing fast energy dissipation and initial crack-interface reconstruction, but networks dominated by hydrogen bonding may show limited long-term strength [11,12]. In contrast, disulfide-bond exchange can reconstruct covalent networks and improve strength recovery, although the exchange process is usually slower and may require external activation [13].

A cooperative dynamic network combining hydrogen-bond reorganization and disulfide-bond exchange can exploit the advantages of both mechanisms. In such a system, hydrogen bonds act as reversible sacrificial interactions that promote interfacial contact, crack-tip passivation, and early-stage healing, whereas disulfide exchange contributes to more permanent network reconstruction and mechanical-property recovery [14,15,16,17,18]. Recent studies have demonstrated the potential of this strategy in self-healing polyurethane adhesives for energetic materials. For example, Cheng et al. [19] reported a disulfide-containing polyurethane adhesive that recovered mechanical properties within 20 min at 60 °C and showed compatibility with TATB. Tu et al. [20] developed a strong self-healing adhesive capable of property recovery within 24 h under ambient conditions and demonstrated improved interfacial performance in a propellant system. Despite this progress, further investigation is still needed to clarify how solid content and energetic components affect the balance between chain mobility, self-healing behavior, and mechanical retention in practical composite formulations.

In this work, a self-healing polyurethane adhesive, denoted PUHD, was rationally designed for energetic-composite applications. PTMEG was used as the soft segment to provide chain flexibility, while IPDI and DHDPS were introduced as hard-segment components to construct hydrogen-bonding sites and dynamic disulfide bonds. The chemical structure, dynamic-bond characteristics, amorphous structure, molecular-weight distribution, and thermal behavior of PUHD were systematically characterized. The self-healing and mechanical properties of neat PUHD were then evaluated, followed by sample filler-containing composites with different solid contents and nitrocellulose/nitroglycerin-based energetic composites. By comparing crack closure, hardness, storage modulus, and healing recovery, this study clarifies the role of the dual dynamic network in damage repair and identifies the trade-off between reinforcement and chain mobility in self-healing energetic composites.

## 2. Experimental Section

### 2.1. Materials

Isophorone diisocyanate (IPDI, 99%), Poly(tetramethylene ether) glycol (PTMEG, Mn = 1000 g⋅mol^−1^), Dibutyltin dilaurate (DBTDL, 95%), 4,4′-Dihydroxydiphenyl disulfide (DHDPS, 98%), Dimethylacetamide (DMAC, ≥99.9%), Tetrahydrofuran (THF, ≥99.9%), Triacetin (TA, ≥95%), Nitrocellulose (NC), Nitroglycerin (NG), and Melamine (C_3_H_6_N_6_, ≥99.5%) were supplied by Xi’an Modern Chemistry Research Institute.

### 2.2. Self-Healing Adhesive Synthesis

**Figure 1:**
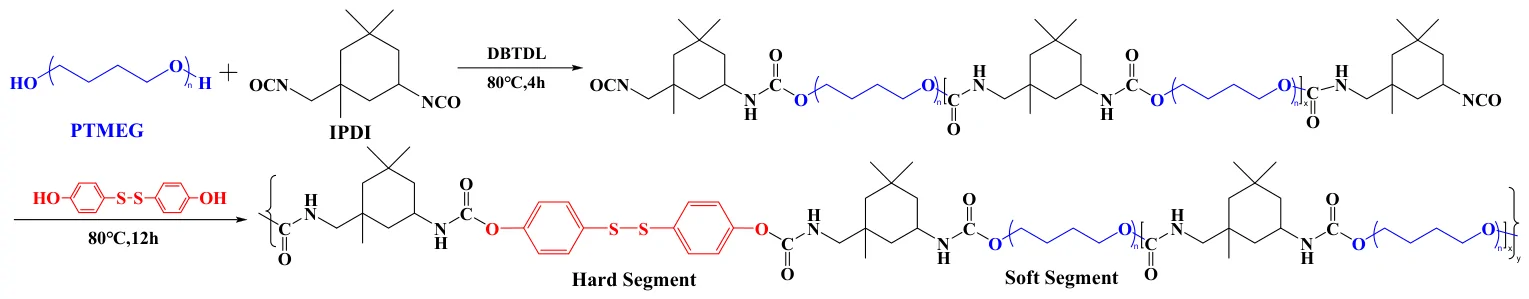
Self-healing adhesive synthesis route.

The self-healing adhesive synthesis pathway is presented in Figure 1 [21]. PTMEG forms the soft segment that provides ductility, while IPDI and DHDPS form the hard segment that offers strength and allows self-healing. The high reversibility of the dynamic disulfide bonds promotes exchange reactions between molecular chains, while the dissociation and reorganization of the weak hydrogen bonds formed by IPDI also contribute to the reconstruction of the molecular network [22]. These two dynamic interactions act synergistically to establish a robust self-healing mechanism, providing sufficient driving force for the material to recover its structural integrity and functional performance after damage [23].

The self-healing adhesive was synthesized in the following two steps. Initially, a mixture of PTMEG (1 eq), IPDI (1.5 eq), and DBTDL catalyst (0.2 wt%) was heated at 80 °C in an oil bath for 4 h with a N_2_ atmosphere to ensure homogeneity. Next, DHDPS was dissolved in DMAC, and the mixture was slowly introduced into the reaction system for further reaction for 12 h while keeping the conditions constant. The slow addition was adopted to avoid localized exothermic effects and to promote uniform chain extension. Following that, the product was placed in a mold and transferred into a vacuum oven for 0.5 h for bubble removal. The molded sample was cured at 80 °C in an oven for 24 h, eventually yielding the yellow PUHD elastomeric product.

### 2.3. Self-Healing Composites Preparation

The effects of component contents on the performance of the self-healing composites were systematically explored using two types of self-healing composites. The first sample was added with solid particles, and the second sample was added with NC/NG energetic premix. Briefly, the dry self-healing adhesive (8.5 g) was dissolved in THF (30 mL). Then, melamine (1 g) and TA (Triacetin; 0.5 g) were added, and the mixture was thoroughly stirred to produce a uniform viscous slurry. Following that, the well-mixed slurry was transferred into a premixing container, which served as the mold, and natural curing and solvent evaporation were allowed for 48 h at ambient temperature. Following complete solvent removal, the cured sample was demolded to obtain the self-healing composite denoted as JQ-a. Samples JQ-b and JQ-c were prepared following the same procedure as JQ-a, with variations only in component proportions. For samples JQ-d~g, a pre-prepared mixture of NC, NG and water was gradually introduced into the homogeneous slurry under gentle stirring. The resulting mixture was then poured into the premixing container, molded, and cured under identical conditions until complete solvent evaporation, after which the samples were demolded. The detailed formulations, summarized in Table 1, were designed to cover a wide range of solid filler and self-healing material PUHD, enabling a comprehensive assessment of their effects on healing behavior and mechanical properties.

### 2.4. Characterization and Testing

The chemical structures of the samples were characterized based on FT-IR spectra detected using an ATR-equipped INVENIO-S spectrometer (Bruker, Bremen, Germany). Data collection involved 16 scans at a resolution of 4 cm^−1^ across a spectral range of 4000 to 500 cm^−1^. Using a 532 nm laser, the Raman spectra were acquired on a LabRAM HR Evolution system (Horiba, Kyoto, Japan) operating with an output power of 50 mW and an integration time of 10 s. The crystalline phases were characterized based on X-ray diffraction (XRD) patterns detected using Cu Kα radiation (λ = 0.1541 nm) on an X-ray diffractometer (Dandong Haoyuan Instruments, Dandong, China). The generator operated at 40 kV and 30 mA, and data were recorded across a scan range of 5° to 55° with 0.05° steps. The molecular weight and its distribution were characterized by 1260 infinity II gel permeation chromatography (Agilent Technologies, Reston, VA, USA). The test was carried out at room temperature with tetrahydrofuran (THF) as the mobile phase at a flow rate of 1.0 mL·min^−1^. The DSC 300 select differential scanning calorimeter (Netzsch, Waldkraiburg, Germany) was used to analyze the thermal transformation behavior of the materials. The test was carried out in nitrogen atmosphere, and the gas flow pattern velocity was 60 mL·min^−1^. About 0.5 mg of the sample was weighed and placed in a closed aluminum crucible. The temperature was raised from 50 °C to 450 °C at a heating rate of 10 °C·min^−1^. To systematically assess self-healing performance, the samples were subjected to cutting and rejoining at room temperature. Then, a high-definition camera was used to observe and record the crack healing. The hardness was measured by Fischerscope HM 2000 nanoindentation instrument (Nantong Fisher Testing Instrument Co., Ltd., Shanghai, China). The dynamic mechanical properties of the materials were tested by Q800 dynamic thermomechanical analyzer (TA instruments, New Castle, DE, USA). The tensile mode was used in the test, and the sample size was 13 mm × 7 mm × 1 mm. The tensile test conditions of variable temperature DMA were as follows: frequency 0.1 Hz, heating rate 5 °C·min^−1^, and temperature range −100 °C to 60 °C. The tensile test conditions of variable frequency DMA are: temperature 20 °C, frequency 0.1 Hz, 1 Hz, and 5 Hz. The storage modulus (*E*′) is automatically recorded by the instrument. To characterize the surface and cross-sectional morphology, the composite samples were subjected to scanning electron microscopy on a ZEISS Sigma 300 system, Jena, Germany. All measurements were performed at least in triplicate to ensure reproducibility, and the reported data represent average values with standard deviations within acceptable ranges. To clarify the healing evaluation procedure, all specimens used for self-healing tests were completely severed before healing rather than subjected to only surface damage. After rejoining the fractured parts, the samples were allowed to heal at room temperature, and all mechanical measurements reported in this work were obtained after a healing time of 48 h. For DMA measurements, tensile loading was applied along the direction normal to the healed interface so that the response mainly reflected the mechanical recovery of the repaired interface. For nanoindentation measurements, the indented locations were selected on the sample surface corresponding to the positions after fracture and subsequent self-healing.

## 3. Results and Discussion

### 3.1. Adhesive PUHD Characterization Analysis

**Figure 2:**
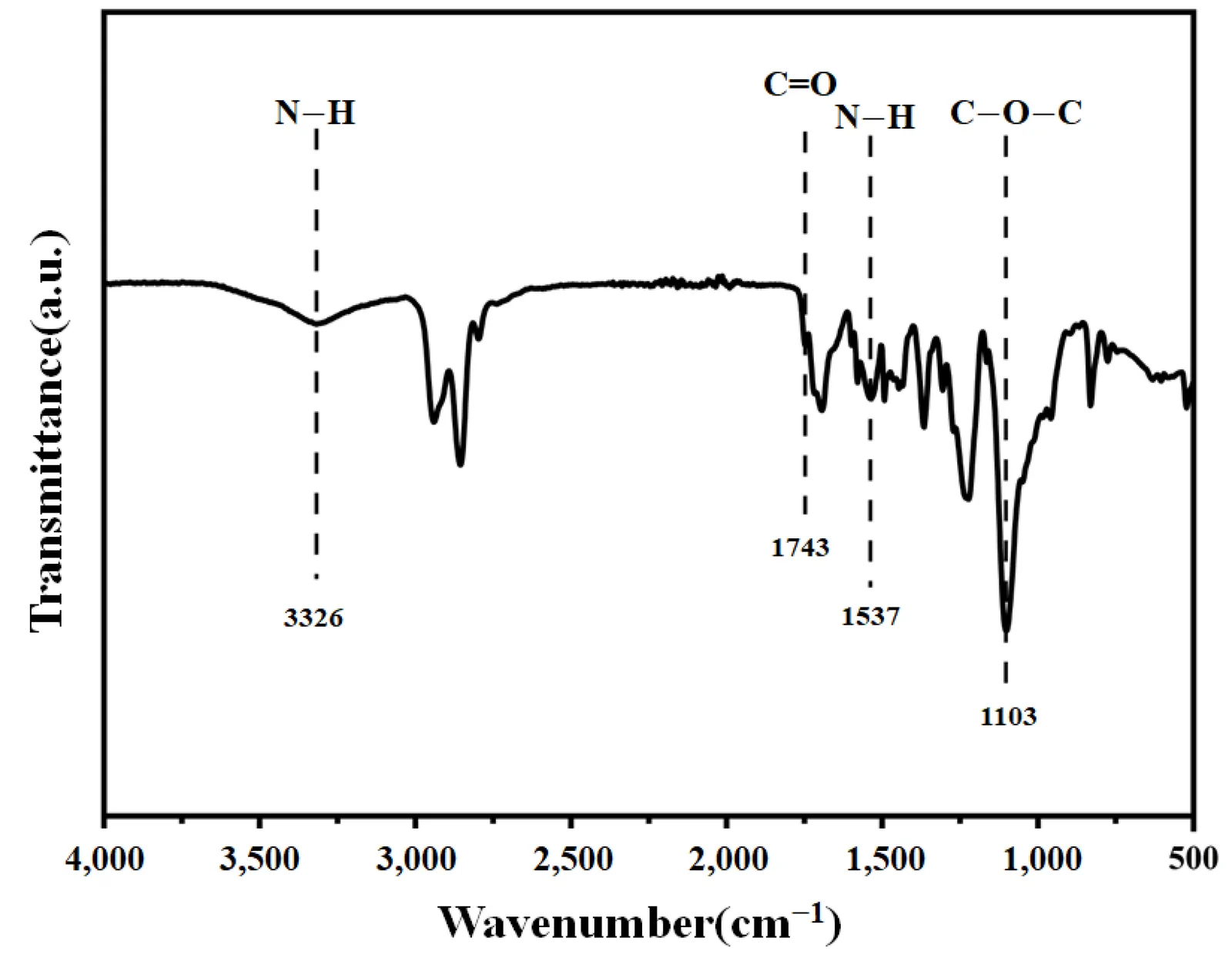
Infrared spectra of PUHD.

The functional groups were characterized by Fourier transform infrared spectroscopy (FT-IR), confirming the presence of relevant chemical groups. As shown in Figure 2, the peaks at 3326 cm^−1^ and 1537 cm^−1^ are ascribed to N-H stretching and bending vibrations, respectively. The peak at 1743 cm^−1^ corresponds to the stretching vibration of carbonyl group (C=O) in carbamate, while the C-O-C stretching leads to a peak at around 1103 cm^−1^. The absence of an -NCO peak at 2265 cm^−1^ confirms its complete reaction and the successful self-healing adhesive synthesis. The Raman results confirm the presence of disulfide-related structural units. The healing behavior is discussed based on reversible interactions and polymer-chain rearrangement.

**Figure 3:**
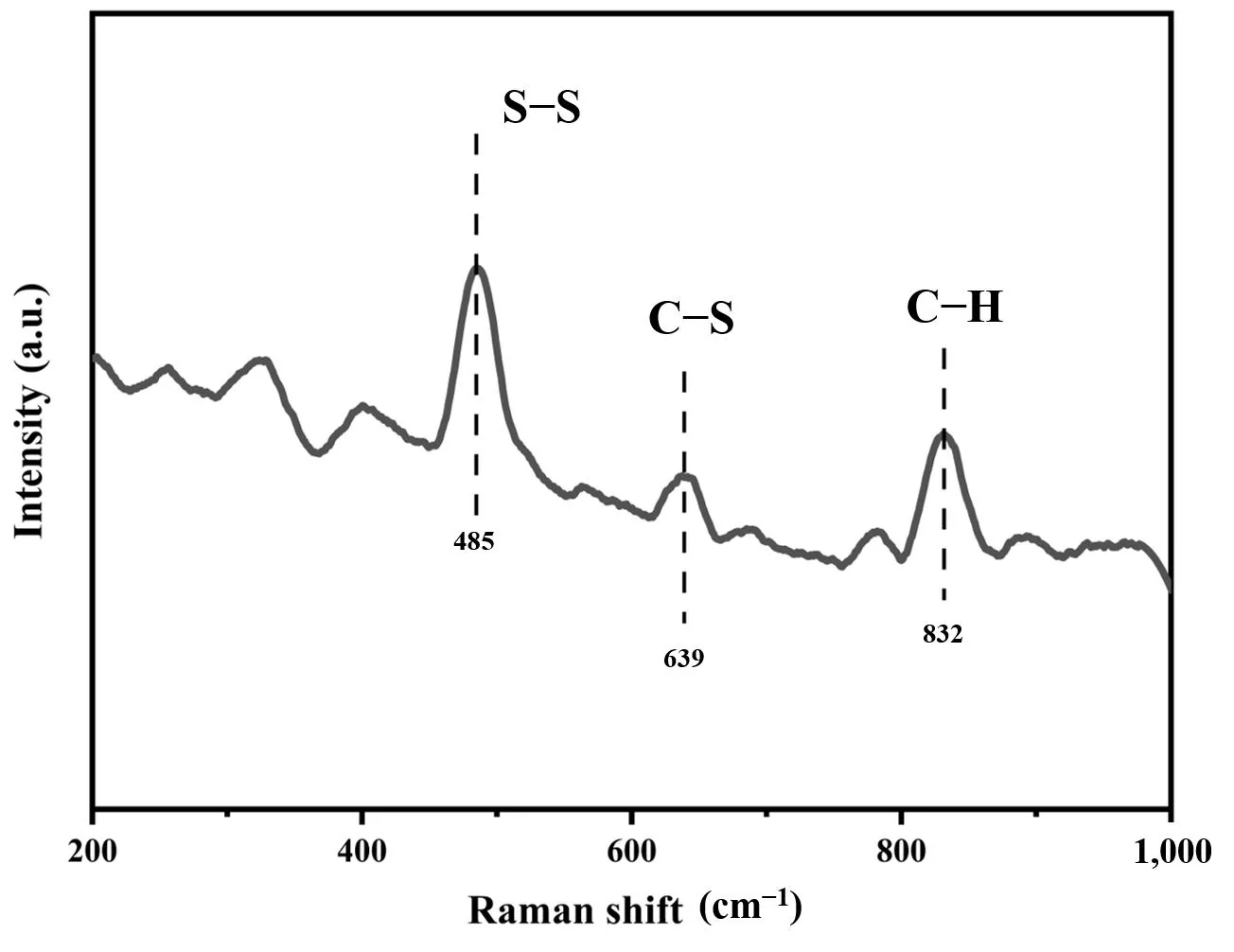
Raman spectra of PUHD.

Raman spectroscopy is highly sensitive to nonpolar bonds and symmetric vibrations, making it suitable for identifying disulfide bonds and aromatic structures and complementing FT-IR in this spectral region. As shown in Figure 3, PUHD exhibits several characteristic Raman bands in the 200~1000 cm^−1^ range. The intense band at 485 cm^−1^ is assigned to the symmetric stretching vibration of S-S bonds, confirming the presence of sulfur-containing dynamic covalent bonds. The band at 639 cm^−1^ is mainly attributed to C-S stretching, possibly coupled with aromatic-ring skeleton vibrations, indicating that sulfur is incorporated into the polymer backbone rather than existing only as S-S linkages. The band at 832 cm^−1^ corresponds to out-of-plane C-H bending of aromatic rings, further verifying the introduction of stable aromatic units.

**Figure 4:**
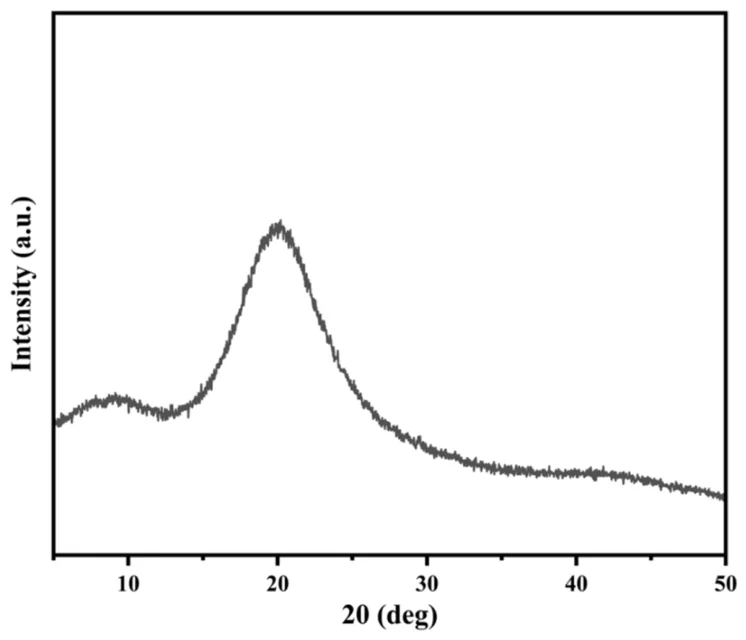
XRD spectrum of PUHD.

The XRD spectrum is shown in Figure 4. A broad diffuse peak centered at approximately 2θ = 20° is observed, whereas no sharp crystalline diffraction peaks appear. This result indicates that PUHD is predominantly amorphous and lacks long-range ordered crystalline domains, although limited short-range ordering may still exist. Such an amorphous structure facilitates the reorganization and exchange of dynamic bonds, including disulfide and hydrogen bonds, thereby providing a structural basis for low-temperature self-healing.

**Figure 5:**
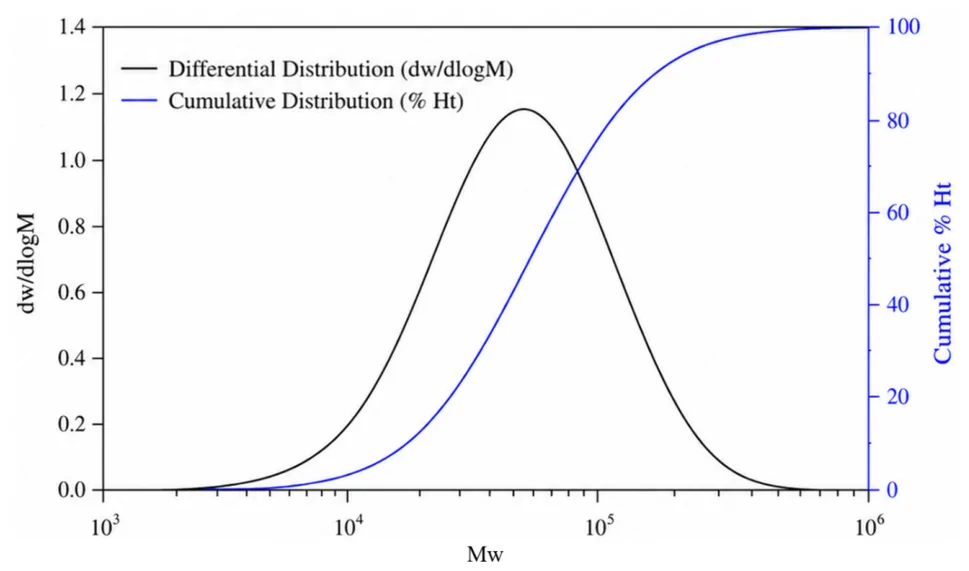
Gel permeation chromatogram of PUHD.

The GPC results of PUHD are presented in Figure 5. The number-average molecular weight (Mn), weight-average molecular weight (Mw), and polydispersity index (PDI) are 36,731 g mol^−1^, 70,677 g mol^−1^, and 1.92, respectively. This molecular weight range provides sufficient chain mobility for reversible-bond recombination after damage while maintaining physical entanglement and structural integrity. The differential molecular-weight distribution (dw/dlogM) is unimodal and relatively symmetric, with the main peak located within 10^4^~10^5^ g mol^−1^, indicating a concentrated molecular-weight distribution. The smooth S-shaped cumulative distribution (%Ht) further suggests uniform molecular-weight fractions. These results describe the molecular-weight characteristics of PUHD, including Mn, Mw and dispersity.

**Figure 6:**
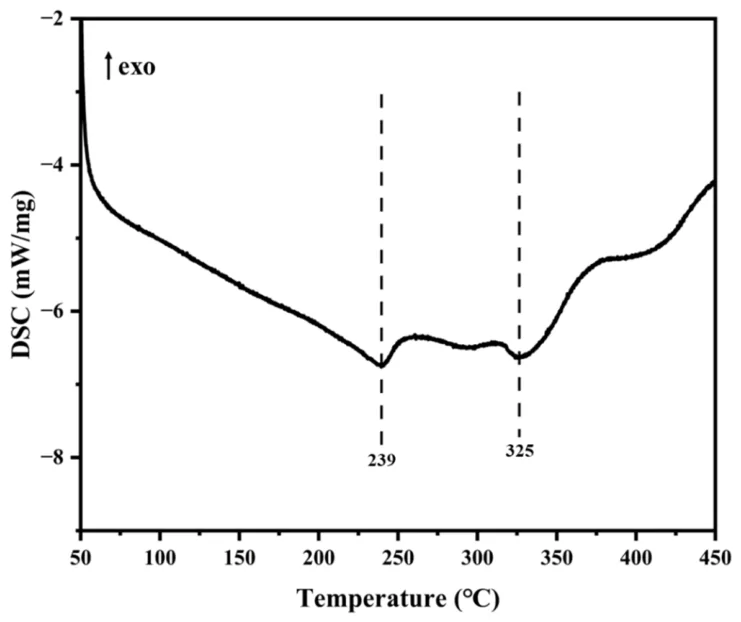
DSC spectrum (50~450 °C) of PUHD.

Figure 6 shows the DSC curve of PUHD over 50~450 °C. The first endothermic peak at approximately 239 °C is associated with the dissociation of locally ordered domains, hydrogen-bonded structures, and microphase rearrangement in the polyurethane network. The second endothermic peak at 325 °C corresponds to the thermal decomposition of hard segments and partial crosslinked networks. No obvious thermochemical transition is observed between 50 and 200 °C, indicating that PUHD has good thermal stability within the temperature range relevant to propellant processing and service.

### 3.2. Surface Crack Self-Healing Behavior

**Figure 7:**
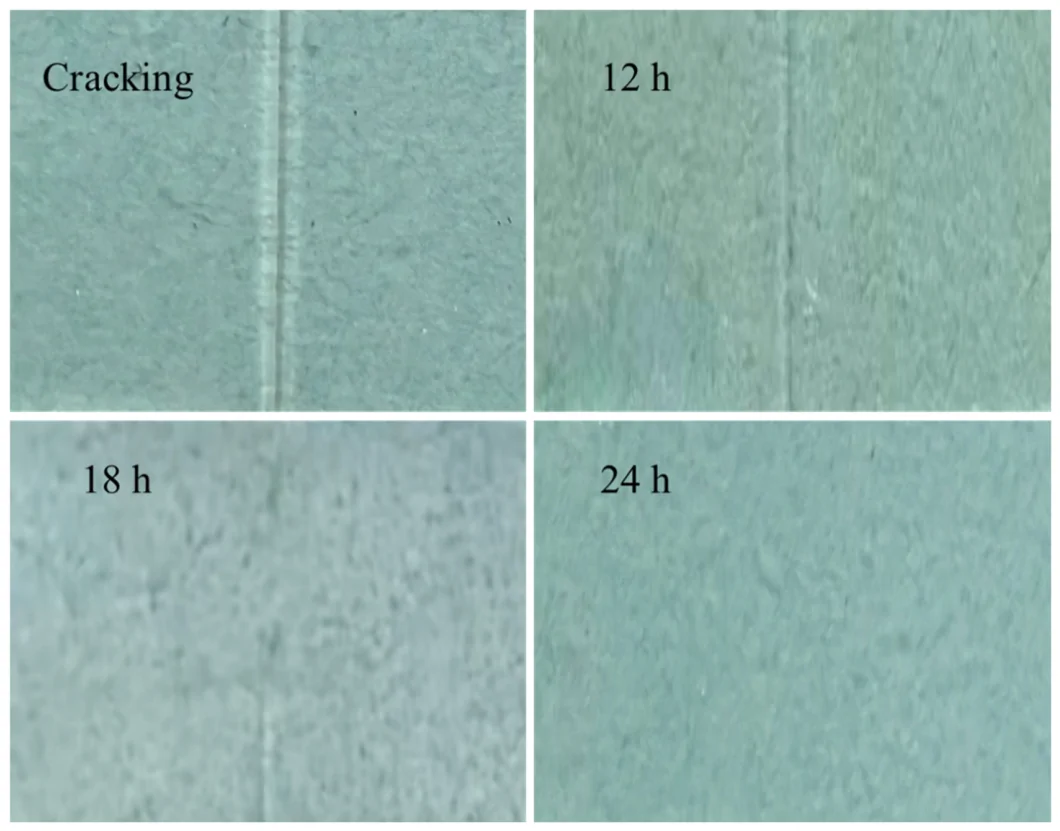
PUHD surface images at cracking and after self-healing 12 h, 18 h and 24 h.

Figure 7 presents images captured over the PUHD healing process. At 12 h following self-healing, PUHD exhibited a significantly reduced number of cracks, which were completely healed after 24 h, confirming that the self-healing adhesive effectively healed the cracks.

**Figure 8:**
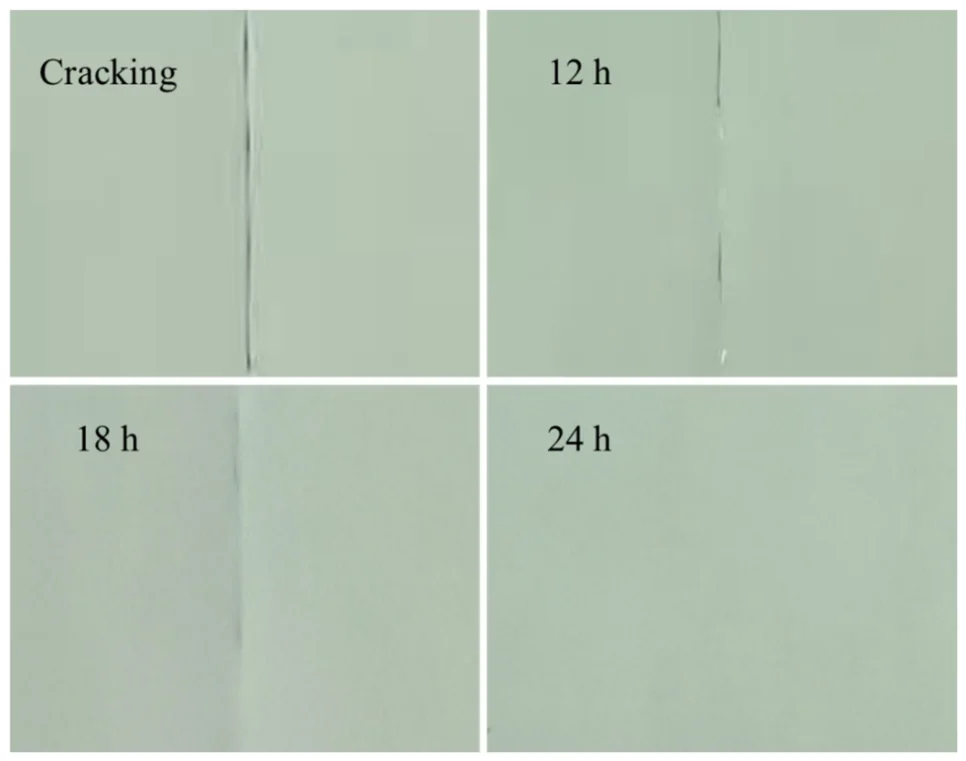
JQ-b surface images at cracking and after self-healing 12 h, 18 h and 24 h.

To confirm that the introduced self-healing adhesive endows the composites with self-healing capacity, we severed them and reassembled the fragments in the mold to observe their self-healing at room temperature. Figure 8 presents photographic documentation of the entire healing process for the JQ-b sample. The crack gradually diminishes, indicating substantial self-healing ability. Quantitative observation reveals that JQ-b shows a significantly reduced number of cracks following 12 h of self-healing, which disappeared completely after 24 h. Therefore, the introduced self-healing adhesive contributed effectively to crack repair. JQ-a~c display significantly lower self-healing performance than the pure adhesive elastomer PUHD. These declines are primarily attributed to the strong interfacial interactions and the large number of solid particles in the composites, which hindered polymer-chain mobility and, in turn, constrained the polymer network rearrangement, resulting in significantly extended self-healing duration. The crack healing of other samples is shown in the attachment.

**Figure 9:**
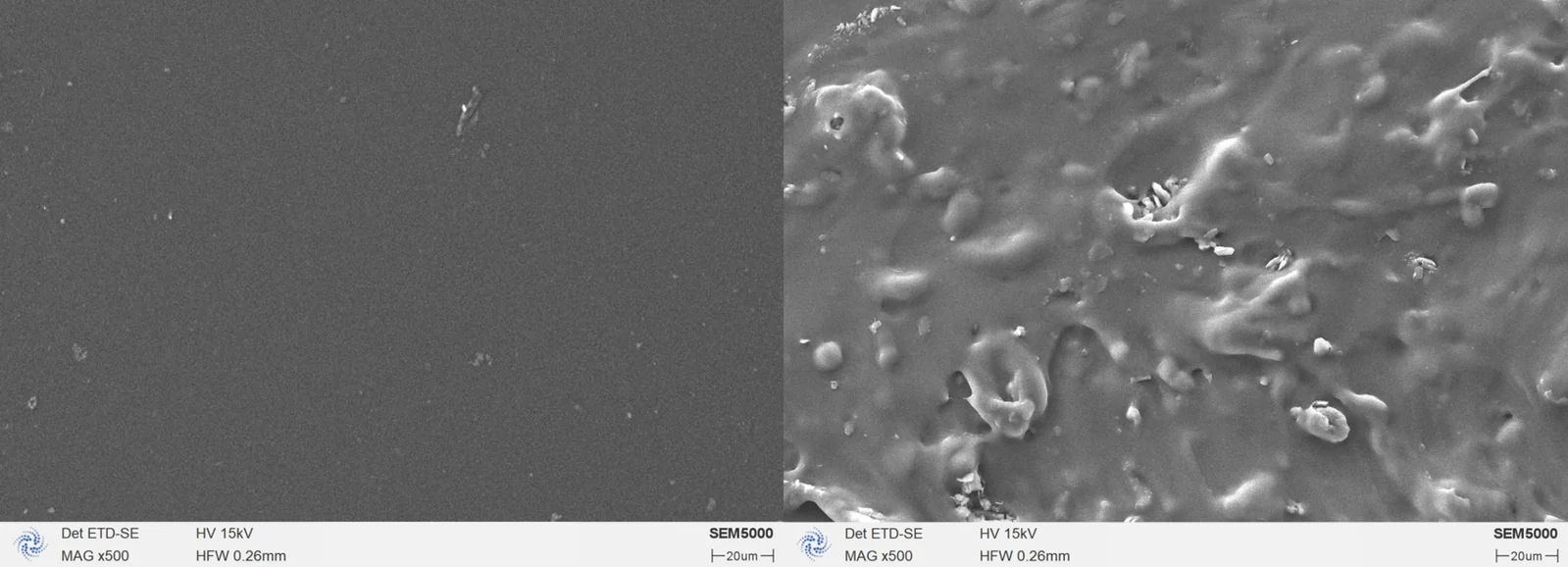
JQ-b surface and fracture cross-section SEM images.

Figure 9 displays SEM images depicting the JQ-b surface and fracture cross-section. The surface of the composite appears relatively smooth, whereas the fracture cross-section demonstrates pronounced heterogeneity, with a clear distinction between the two regions. The solid particles that hinder the self-healing of the composites can be directly observed on the uneven fracture surface.

### 3.3. Effects of Composition on Mechanical Properties

**Figure 10:**
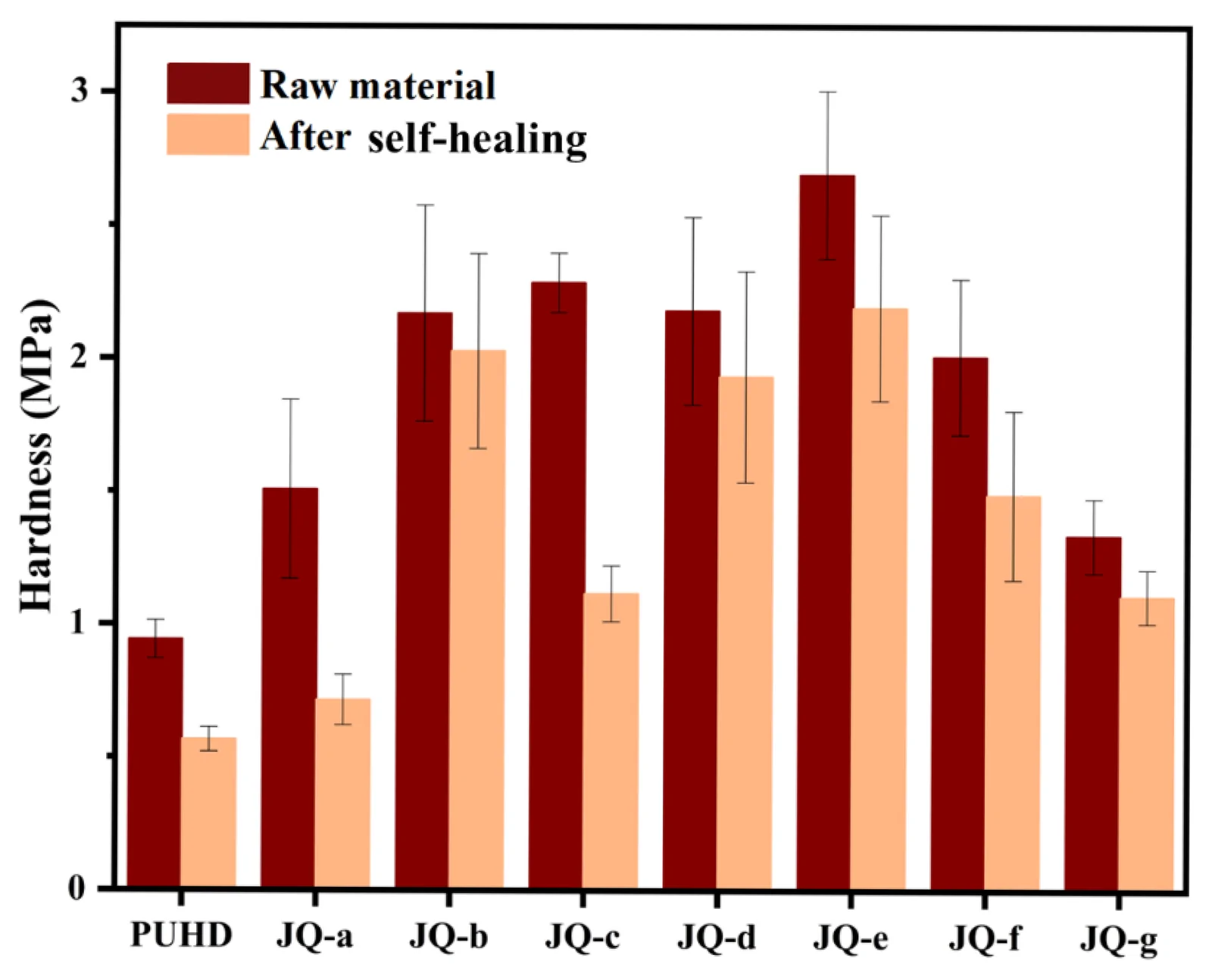
Hardness comparison diagram of samples at pristine state and after self-healing.

Figure 10 compares the hardness of the original and healed samples. PUHD shows the lowest initial hardness of 0.94 MPa. For JQ-a~JQ-c, increasing the melamine content from 10 to 40 wt% raises the initial hardness from 1.53 to 2.28 MPa, demonstrating the reinforcing effect of the rigid filler. The simultaneous decrease in the PUHD fraction and increase in interfacial constraints also restrict chain diffusion, which accounts for the lower recovery at excessive filler loading. The JQ-d~JQ-g trend is non-monotonic because energetic-phase content, continuous-binder fraction and chain mobility change concurrently. JQ-e exhibits the highest initial hardness (2.69 MPa), consistent with an intermediate composition that retains a sufficiently continuous PUHD phase while benefiting from reinforcement by the non-PUHD components. Further increases in NC/NG content reduce the continuous load-bearing PUHD network, and the initial hardness decreases to 1.99 MPa for JQ-f and 1.35 MPa for JQ-g. These results support a qualitative multi-factor explanation rather than attribution of the trend to a single component.

**Figure 11:**
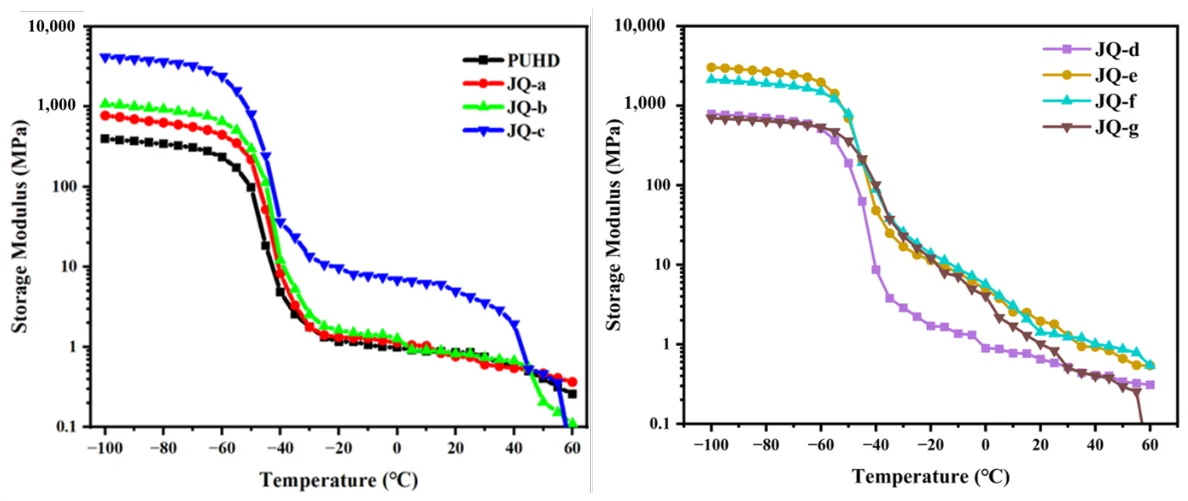
Storage modulus curves of samples (−100 °C~60 °C).

As shown in Figure 11, all samples maintain a high storage modulus in the low-temperature region, followed by a pronounced decrease near −40 °C, indicating glass transition in this temperature range. The storage modulus of JQ-c is higher than those of JQ-a and JQ-b in the low-temperature and transition regions, while the curves gradually converge and cross at the high-temperature region. JQ-e and JQ-f exhibit relatively high moduli and therefore greater rigidity, whereas JQ-d shows a lower modulus and weaker load-bearing capacity. The more stable modulus retention of JQ-e and JQ-f upon heating suggests better overall mechanical performance.

**Figure 12:**
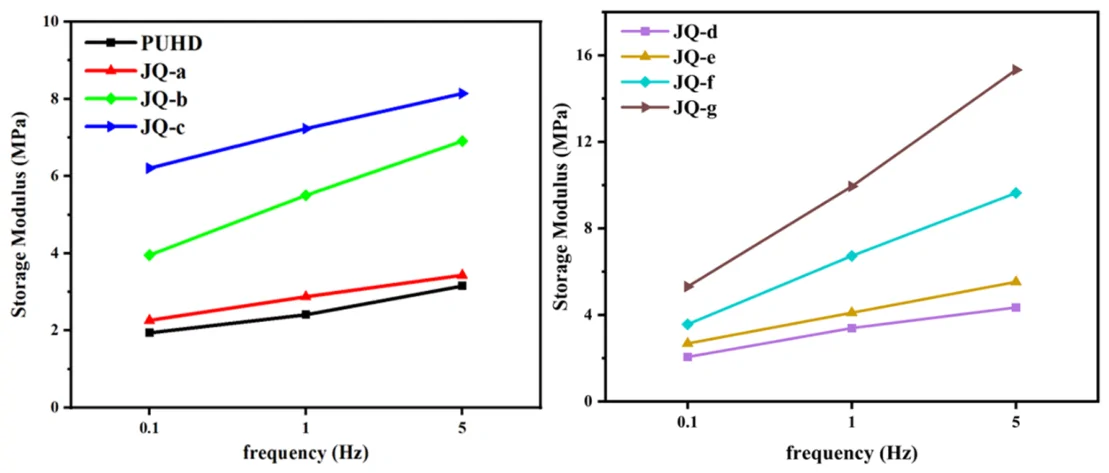
Storage modulus of pristine samples at different frequencies (0.1 Hz, 1 Hz, 5 Hz).

Figure 12 shows the effect of loading frequency on the tensile storage modulus of the pristine samples. As the frequency increases from 0.1 to 5 Hz, the storage modulus of all samples increases, indicating a stronger elastic response under high-frequency loading. At 5 Hz, JQ-c containing 40% solid filler reaches a storage modulus of 8.14 MPa, whereas PUHD without filler exhibits only 3.15 MPa, confirming that filler incorporation increases the overall modulus. As the PUHD content decreases, the storage modulus further increases, and this enhancement becomes more pronounced at higher frequencies; JQ-g reaches 15.33 MPa.

**Figure 13:**
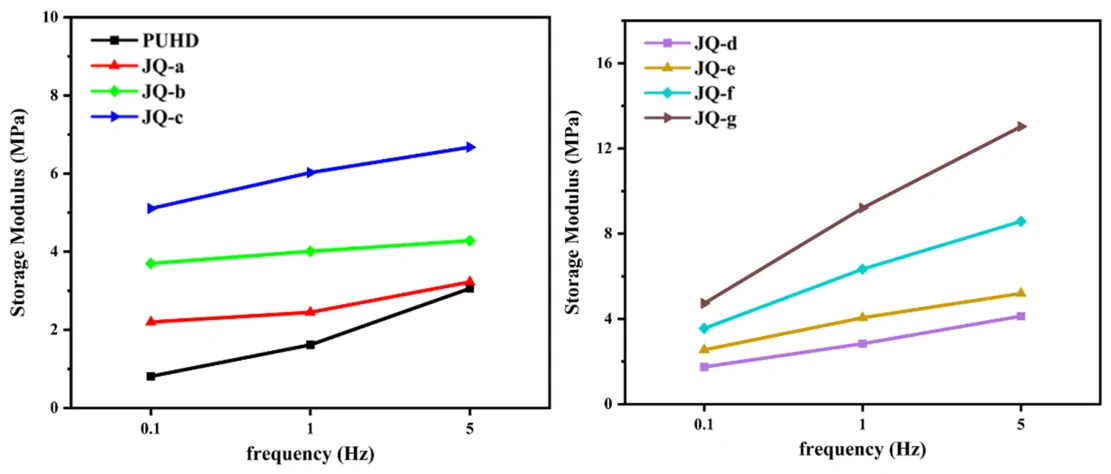
Storage modulus of healed samples at different frequencies (0.1 Hz, 1 Hz, 5 Hz).

Figure 13 illustrates the frequency-dependent tensile storage modulus of the healed samples. After healing, the storage modulus still increases with frequency, indicating that the healed materials retain a clear frequency-dependent response. However, the modulus values are lower than those of the pristine samples, suggesting that the internal network and interfaces are reconnected but not fully restored to their original integrity. The modulus ranking remains essentially unchanged at different frequencies, confirming that the rigidity contributed by the solid filler is retained after healing.

### 3.4. Healing Efficiency Based on Hardness and Storage Modulus

The self-healing efficiency, *E_H_*, is defined herein to quantify the self-healing performance:

(1) $$E_{H}\;\left(\%\right)=\;\left(\frac{hardness\;of\;healed\;sample}{hardness\;of\;original\;sample}\right)\times \;100\%$$

**Figure 14:**
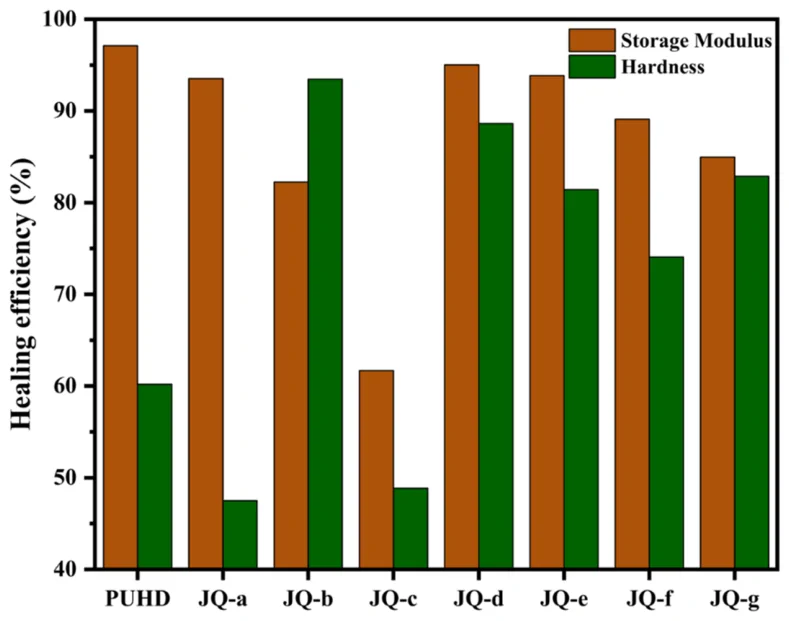
Samples’ healing efficiency of storage modulus and hardness.

Figure 14 compares the healing efficiencies calculated from storage modulus and hardness using the same averaged pristine and healed datasets for all samples. The storage-modulus/hardness recovery values are approximately 97%/60% for PUHD, 94%/48% for JQ-a, 82%/94% for JQ-b, 62%/49% for JQ-c, 95%/88% for JQ-d, 94%/82% for JQ-e, 89%/74% for JQ-f, and 85%/83% for JQ-g. JQ-d therefore shows slightly higher percentage recovery than JQ-e. However, percentage recovery alone does not represent the absolute post-healing load-bearing level: JQ-e has an initial hardness of 2.69 MPa and retains 2.19 MPa after healing, whereas JQ-d starts at 2.22 MPa and retains approximately 1.95 MPa. Accordingly, JQ-e is the NC/NG-containing formulation with the highest initial and post-healing hardness. These results indicate that JQ-b, JQ-d, JQ-e, and JQ-g retain favorable recovery levels in both storage modulus and hardness.

## 4. Conclusions

A self-healing polyurethane adhesive (PUHD) based on synergistic hydrogen-bond reorganization and disulfide-bond exchange was developed and evaluated for energetic-composite applications. Structural characterization confirmed the successful formation of urethane/urea groups and dynamic disulfide bonds, while XRD and GPC indicated a predominantly amorphous polymer network with an appropriate molecular-weight distribution for dynamic-bond reorganization. DSC results further showed no obvious thermal transition below 200 °C, suggesting suitable thermal stability for processing and service conditions. Neat PUHD exhibited visible crack closure within 24 h at ambient temperature, with a hardness of 0.94 MPa and a storage modulus of 3.15 MPa at 20 °C and 5 Hz; its storage-modulus recovery reached approximately 97% after healing. The introduction of the solid filler increased hardness and storage modulus but restricted polymer-chain mobility, thereby slowing the healing process and lowering recovery when the solid content was excessive. In the energetic composites, JQ-d showed slightly higher percentage recovery, whereas JQ-e retained the highest absolute hardness after healing (2.19 MPa) and better self-healing efficiency of hardness (82%) and storage modulus (94%), showing a good balance between mechanical properties and self-healing properties. Overall, the dual dynamic PUHD network provides an effective molecular-design strategy for improving the intrinsic damage tolerance and mechanical reliability of energetic composites, while the solid-content-dependent results highlight the need to balance reinforcement with polymer-chain mobility in future formulation optimization.

## Figures and Tables

**Table 1:** The detailed formula of the samples.

Sample	Component Name and Proportion			
	NC/NG Premix (Total)	Solid Filler (Melamine)	PUHD	TA
JQ-a	0	10%	85%	5%
JQ-b	0	25%	70%	5%
JQ-c	0	40%	55%	5%
JQ-d	20%	20%	50%	10%
JQ-e	30%	20%	40%	10%
JQ-f	40%	20%	30%	10%
JQ-g	50%	20%	20%	10%

Note: NC and NG denote nitrocellulose and nitroglycerin, respectively. The listed values represent the total content of a fixed-ratio NC/NG premix; the internal NC/NG ratio was unchanged across JQ-d~JQ-g.

## Data Availability

The original contributions presented in this study are included in the article. Further inquiries can be directed to the corresponding author.
